# Update on biomarkers in systemic sclerosis: tools for diagnosis and treatment

**DOI:** 10.1007/s00281-015-0506-4

**Published:** 2015-07-14

**Authors:** Alsya J. Affandi, Timothy R. D. J. Radstake, Wioleta Marut

**Affiliations:** Department of Rheumatology and Clinical Immunology, University Medical Center Utrecht, Heidelberglaan 100, 3584 CX Utrecht, The Netherlands; Laboratory of Translational Immunology, University Medical Center Utrecht, Heidelberglaan 100, 3584 CX Utrecht, The Netherlands

**Keywords:** Autoantibodies, Biomarker, miRNAs, Pulmonary fibrosis, Skin fibrosis, Systemic sclerosis

## Abstract

Systemic sclerosis (SSc) is a complex autoimmune disease in which immune activation, vasculopathy, and extensive fibrosis of the skin and internal organs are among the principal features. SSc is a heterogeneous disease with varying manifestations and clinical outcomes. Currently, patients’ clinical evaluation often relies on subjective measures, non-quantitative methods, or requires invasive procedures as markers able to predict disease trajectory or response to therapy are lacking. Therefore, current research is focusing on the discovery of useful biomarkers reflecting ongoing inflammatory or fibrotic activity in the skin and internal organs, as well as being predictive of future disease course. Recently, remarkable progress has been made towards a better understanding of numerous mechanisms involved in the pathogenesis of SSc. This has opened new possibilities for the development of novel biomarkers and therapy. However, current proposed biomarkers that could reliably describe various aspects of SSc still require further investigation. This review will summarize studies describing the commonly used and validated biomarkers, the newly emerging and promising SSc biomarkers identified to date, and consideration of future directions in this field.

Systemic sclerosis (SSc) is an autoimmune disease characterized by fibrosis of the skin and internal organs, preceded by vascular and immune dysfunction [1]. Depending on the extent of cutaneous fibrosis, SSc is classified into two major subtypes: limited cutaneous (lcSSc) and diffuse cutaneous SSc (dcSSc). In lcSSc, skin thickening is restricted to the areas distal to the elbows and/or knees, such as hands and fingers. In dcSSc, the presence of skin lesions is more extensive and internal organs involvement is relatively more severe. This classification is supported by the association with specific autoantibodies that specifically define the two types of clinical phenotypes. Both SSc phenotypes can be complicated by severe internal organ dysfunction. Pulmonary fibrosis and pulmonary arterial hypertension (PAH) are the two most feared complications, representing the major causes of mortality in SSc patients [2]. Owning to its complex nature and heterogeneity, SSc remains one of the greatest challenges to both investigators and physicians. Despite intense investigation, so far, only a few biomarkers for SSc have been fully validated and widely accepted. Herein, we present a review of the literature on promising prognostic biomarkers, biomarkers of disease activity, skin fibrosis, and lung involvement, with the aim to provide a comprehensive update on usability of biomarkers for research and clinical guidance.

## Diagnostic and prognostic biomarkers

### SSc-specific autoantibodies as predictive markers

The presence of autoantibodies is a central defining aspect of autoimmune diseases. Autoantibodies are seen at the first diagnosis in more than 95 % of SSc patients and have been associated with distinct disease subtypes and with differences in disease severity. Antitopoisomerase I (ATAs) and anticentromere antibodies (ACAs) are the most widely used diagnostic biomarkers for SSc [3–5].

Anti-Scl-70 antibodies originally identified by Douvas et al. [6] are directed against DNA topoisomerase I [7] and therefore should be more accurately termed antitopoisomerase I antibodies (ATAs). These autoantibodies are seen predominantly in dcSSc patients; however, their presence is not entirely restricted to this clinical subset since a subgroup of lcSSc patients was also found to be ATA-positive [8, 9]. ATA has been associated with poorer prognosis, increased mortality, pulmonary fibrosis, and cardiac involvement [9–12]. Another recent study of clinical outcomes in patients with digital ulcers showed that patients positive for ATAs developed Raynaud’s phenomenon earlier and had double rate of lung fibrosis as compared with ACA-positive patients [13]. Some reports indicate that changes in ATA titers over time can be useful in monitoring disease activity and progression and therefore useful for prognostic purposes [14].

ACAs recognize centromeric protein from CENP-A to CENP-F, of which CENP-B is reported to be a major autoantigen reacting with virtually all anti-CENP-positive SSc sera [15, 16]. ACAs are found in 20 to 30 % of SSc patients, and in up to 90 % of lcSSc patients [4, 17]. In patients with Raynaud’s phenomenon, ACAs have been reported to predict the onset of lcSSc [3, 18]. While severe interstitial fibrosis and renal crisis occur rarely, pulmonary arterial hypertension occurs in about 20 % of anti-CENP patients [9, 10, 19]. Anti-CENPs are often associated with other antibodies, such as anti-Sjogren’s-syndrome-related antigen A (anti-Ro) [20] or antimitochondrial antibodies [21]. Moreover, it has been reported that ACA positivity correlated with a more favorable prognosis and lower mortality compared with the positivity of other SSc-related autoantibodies [22].

Antibodies against RNA polymerase I and III (anti-RNP I and III) are detected with high specificity in SSc patients (98–100 %) [23, 24]. Their prevalence varies from 10 to 25 % in different SSc cohorts. Anti-RNP I and III are associated with dcSSc involvement and renal crisis [25]. More recently, it has been shown that the presence of anti-RNP is associated with rapid onset of the disease and skin thickening. Therefore, they are still among the best predictive markers available for rapid skin progression [26]. Autoantibodies to RNP II are uncommon and are not specific for SSc since they can be also be found in the sera of systemic lupus erythematosus (SLE) and overlap syndrome [27].

Additional SSc-specific autoantibodies with diagnostic or prognostic utility include the anti-Th/To and anti-U3 RNP (antifibrillarin) antibodies. Th/To autoantibodies are directed against subunit of RNase P and RNase MRP [28]. They are found in 2–5 % of SSc patients and are clinically associated with lcSSc (8.4 % of lcSSc patients, 0.6 % of dcSSc) [10, 29]. Among lcSSc patients, anti-Th/To are a marker of the worst survival rate perhaps related to severe pulmonary embolism (PE) preceding PAH and renal crisis [19]. It has been reported that the presence of anti-Th/To may assist in identifying sine SSc subset in patients with pulmonary fibrosis [30]. Anti-U3 RNP antibodies target fibrillarin, a small protein belonging to the U3 small nuclear ribonucleoprotein (RNP) complex. Although they are considered to be a specific marker for SSc, they are found in less than 7 % of SSc sera and their confirmation using advanced techniques continues to be a challenge [31]. They are most frequent in males and African Americans with SSc and are associated with muscle involvement and increased risk of PAH [32].

Recently, autoantibodies against angiotensin II type 1 receptor (AT_1_R) and endothelin-1 type A receptor (ET_A_R) have been shown to be elevated in the sera of most SSc patients, and associated with vascular and fibrotic complications [33, 34]. They are more frequent in SSc-PAH/connective tissue disease-PAH compared to other forms of pulmonary hypertension. Therefore, they could serve as new predictive and prognostic biomarkers of PAH in SSc. These autoantibodies not only predict development of PAH but also are associated with higher mortality in SSc patients [33].

More recently, antiestrogen receptor antibodies (anti-ERα) were detected in sera of 42 % SSc patients; whereas, no anti-ERα antibodies were found in healthy controls. Anti-ERα antibodies were significantly associated with disease activity and were mainly found among patients with the diffuse form of the disease, the ANAs positivity, and the late capillaroscopy pattern. However, it is important to note that anti-ERα antibodies are not specific for SSc since they were also detected in patients with SLE but not in other patients with other autoimmune diseases such as rheumatoid arthritis (RA) or Behcet’s disease [35].

Other autoantibodies of relevance to SSc but less commonly present in SSc patients include the anti-U11/U12 RNP antibodies. They are highly associated with severe lung fibrosis, and anti-PM-Scl antibodies found in patients with SSc overlap and with myositis [36]. Remarkably, many of the autoantibodies in SSc show correlation with other type of biomarkers such as those for skin fibrosis and pulmonary complications, as we will discuss below (Table 1).

**Table 1:** Diagnostic and prognostic biomarkers—autoantibodies

Biomarker	Source	Association	Reference
Antitopoisomerase I (ATAs)	Serum	dcSSc, poor prognosis, increased mortality, lung fibrosis, cardiac involvement	[8–14]
Anticentromere (ACAs)	Serum	lcSSc, PAH, more favorable prognosis, lower mortality	[4, 9, 10, 15–17, 19]
Anti-RNA polymerase I and III (anti-RNAP I, III)	Serum	dcSSc, skin progression, renal crisis	[23–26]
Anti-Th/To	Serum	lcSSc, a marker of worst survival rate, muscle involvement, PAH	[10, 19, 29, 30]
Anti-U3 RNP	Serum	Activity	[31, 32]
Anti-AT_1_R, anti- ET_A_R	Serum	Activity, PAH, vascular and fibrotic complications, higher mortality	[33]
Anti-ERα	Serum	Activity, dcSSc	[35]
Anti-U11/U12 RNP	Serum	Severe lung fibrosis	[36]

### Circulating miRNA

miRNAs are a class of endogenous and evolutionary conserved short, noncoding RNAs that bind to the 3′ untranslated region of target genes. Once bound, miRNAs repress target gene translation or promote mRNA destabilization and degradation. They are expressed in a tissue-specific and cell type-specific manner but can also circulate in the bloodstream, and such circulating miRNAs are remarkably stable [37]. This has raised the possibility that miRNAs may be probed in the circulation and can serve as novel diagnostic markers. It has been shown that the elevated expression of pro-fibrotic miRNAs and reduced expression of antifibrotic miRNAs are important factors in the developments of fibrosis in SSc. Furthermore, several studies have already demonstrated that the levels of selected miRNAs were altered in the serum of SSc patients [38, 39].

The levels of miR-150 were found to be downregulated in the serum of SSc patients versus healthy controls and were correlated with more severe clinical manifestations. For instance, higher incidence of antitopoisomerase I antibodies and a higher prevalence of pitting scars were seen in patients with lower miR-150 levels than in those without. Although the difference did not reach statistical significance, patients with lower miR-150 levels had a higher ratio of dcSSc to lcSSc and a higher modified Rodnan skin score (mRSS) when compared with patients with normal levels of miR-150 [40]. Similar correlation was observed for another miRNA—miR196a, where patients with lower serum miR-196a levels had significantly higher ratio of dcSSc to lcSSc and higher mRSS; in addition, they showed higher prevalence of pitting scars than those without [41]. Other studies evidenced that the serum level of miR-30b [42] and let-7a [43] were highly decreased in SSc patients versus healthy controls. Both miRNAs were again downregulated more strongly in diffuse subset of SSc than in limited SSc. Interestingly, miR-30b and let-7a were inversely correlated with mRSS [42, 43].

On the contrary, serum levels of miR-92a [44] and miR-142-3p [45] were markedly higher in SSc when compared to healthy controls or SLE, dermatomyositis, and scleroderma spectrum disorder (SSD) patients. Therefore, these miRNAs may provide as useful diagnostic markers for the differentiation of SSc from other scleroderma spectrum disorders (Table 2).

**Table 2:** Prognostic biomarkers—circulating miRNA

Biomarker	Expression in SSc	Source	Association	Reference
miR-150	Downregulated	Serum	dcSSc, skin fibrosis	[40]
miR196a	Downregulated	Serum	dcSSc, skin fibrosis, higher prevalence of pitting scars	[41]
miR-30b	Downregulated	Serum	dcSSc, inversely correlated with skin fibrosis	[42]
let-7a	Downregulated	Serum	dcSSc, inversely correlated with skin fibrosis	[43]
miR-92a	Upregulated	Serum	SSc	[44]
miR-142-3p	Upregulated	Serum	SSc	[45]

## Biomarkers for disease activity

One of the main challenges of SSc studies is to develop a sufficient tool for a global measurement for disease activity that represents an ongoing disease activity and/or response to treatment. Unlike other autoimmune diseases such as SLE or RA, for many SSc patients, ongoing inflammation is difficult to assess and vascular and tissue fibroses are not easy to quantitate especially in the early stage of the disease. Currently, the Valentini disease activity index, developed by The European Scleroderma Study Group (EScSG), is the most widely used activity score in SSc studies [46]. This activity index includes mRSS, DLCO, and ESR, without a specific biochemical marker. The Medsger disease severity scale is also frequently used as a measure of disease activity [47]. However, this assessment may more reflect damage or severity rather than the ongoing disease activity.

The enhanced liver fibrosis (ELF) test was developed as a clinical grade serum test for chronic liver diseases, including procollagen-III aminoterminal-propeptide (PIIINP), tissue inhibitor of matrix metalloproteinase-1 (TIMP-1), and hyaluronic acid (HA) in its algorithm. Each of these three serum markers is increased in SSc patients as compared to healthy controls and associated with more severe complications or increased mortality [48–50]. Recently, ELF test was tested in SSc patients and showed significant correlations with both disease activity and severity [51]. ELF score also correlated with mRSS, Health Assessment Questionnaire-Disability Index (HAQ-DI), and inversely correlated with DLCO, but it did not correlate with vasculopathy features such as PAH [51].

Other candidate biochemical markers for disease activity and severity in SSc have been derived from their association with an organ-specific involvement. The markers for pulmonary involvement in SSc, serum vWF and KL6, were significantly associated with disease severity and activity, respectively [52, 53]. The serum level of cartilage oligomeric matrix protein (COMP), a molecule that has been associated with skin and lung fibrosis as we describe below, also showed correlation with disease severity [54]. The angiopoietin/Tie2 axis has gained some interests due to their roles in angiogenesis. Serum levels of angiopoietin-2 (Ang-2), but not angiopoietin-1, have been shown to correlate with disease activity [55]. Another study found a similar but not significant trend in plasma, although the authors found a stronger correlation using the ratio of Ang-2 and its soluble receptor Tie2 with disease activity [56].

The classic inflammatory cytokine IL-6 is increased in the sera of SSc patients and has been associated with multiple organ involvement including skin [57], the occurrence of pulmonary fibrosis [58], FVC decline, and increased mortality [59]. Plasma IL-6 level was found to be higher in ATA-positive and anti-RNAP III-positive patients but not in ACA-positive SSc patients [60]. In one study, serum IL-6 was shown to correlate with disease activity [61], although this was not found by others [62]. In a genetic association study, IL-6 polymorphism in SSc patients was shown to be associated with disease activity and HAQ-DI, unfortunately circulating IL-6 was not measured [63].

Growth differentiation factor 15 (GDF-15) is a distant member of the TGFβ superfamily and found to be elevated in the serum of SSc patients compared to healthy controls [64, 65]. In SSc patients, serum GDF-15 levels showed strong correlation with mRSS, disease activity, and disease severity [65], in particular those with pulmonary involvement, as we will discuss below.

It is important to note that many of these studies assessing disease activity are cross-sectional and limited to small cohorts at single centers. Future multicenter validation and longitudinal study are necessary to assess their sensitivity for changes over time in a larger population. Multibiomarker approach such as the ELF score should also be considered (Table 3).

**Table 3:** Biomarker in SSc disease activity

Biomarker	Source	Association	Reference
ELF test	Serum	Activity, severity	[51]
vWF	Serum	Severity	[52]
KL-6	Serum	Activity	[53]
Ang-2	Serum	Activity	[55]
COMP	Serum	Activity	[54]
IL-6	Serum	Activity	[61, 62]
GDF-15	Serum	Activity, severity	[65]

## Biomarkers correlating with skin fibrosis

Skin fibrosis, the hallmark of SSc, is defined as an excess deposition and accumulation of extracellular matrix in the dermis. Despite our growing understanding of this process and many available targets, our therapeutic success in ameliorating skin fibrosis in SSc is still minimal. Even today, the gold standard for measuring SSc skin fibrosis is mRSS, a relatively simple determination of skin thickness, which has significant inter-observer variability and is rather subjective. Moreover, the mRSS may not be sensitive enough to find smaller but important and early changes in skin thickening [66]. Therefore, there is a need for other specific and more precise markers for assessing skin fibrosis.

### Peripheral blood biomarkers

There is a large number of potential circulating biomarkers for skin fibrosis which include COMP, MMP-9, MMP-12, LOX, IL-6, IL-10, and CXCL4 (Table 4). Here, we will discuss those biomarkers that are most robustly shown to be of potential relevance.

**Table 4:** Emerging biomarker in SSc skin fibrosis

Biomarker	Source	Association	Reference
COMP	Skin, serum	dcSSc, skin fibrosis	[54, 67–69]
MMP-9, MMP-12	Skin, serum	dcSSc, skin fibrosis	[75, 78, 79]
LOX	Skin, serum	dcSSc, skin fibrosis	[80]
IL-6, IL-10	Serum	Skin fibrosis	[57, 62]
CXCL4	Serum	dcSSc, skin fibrosis	[81]
TSP-1, IFI44, Siglec-1	Skin	Skin fibrosis	[83]
LH2	Skin	Skin fibrosis activity	[84]

Cartilage oligomeric protein 1 (COMP) is a non-collagenous glycoprotein, mostly synthesized by chondrocytes, osteoblasts, tenocytes, synovial fibroblasts, and dermal fibroblasts. This protein, highly regulated by TGF-β, is not detectable in the healthy skin but is highly overexpressed in skin biopsies and fibroblasts of SSc patients [67, 68]. Moreover, COMP was found to be increased in SSc sera and correlated with the extent of skin involvement, as assessed by mRSS and ultrasound [69]. More recent study confirmed high levels of COMP in the serum of SSc patients, and its level was higher in dcSSc subset than in lcSSc [54].

Matrix metalloproteinases (MMPs), responsible for the degradation of collagens and other extra cellular matrix (ECM) proteins, are also involved in the release and activation of many cytokines and growth factors [70]. Several inhibitors are known to control their activity. Both, MMPs and their inhibitors, were extensively studied in the pathogenesis of SSc. MMP-9 and MMP-12 were found to be a potential markers for skin fibrosis.

MMP-9, whose substrates include type IV collagen in basement membrane, has been associated with chronic inflammatory autoimmune diseases, including rheumatoid arthritis [71] and SLE [72]. Moreover, its overexpression has been reported in various pathologic conditions characterized by excessive fibrosis, including idiopathic pulmonary fibrosis [73] and chronic pancreatitis [74]. In SSc, fibroblasts isolated from SSc patients expressed more MMP-9 than healthy controls. Furthermore, serum level of MMP-9 was elevated in SSc, with higher concentration in dcSSc compared to lcSSc, and correlated well with mRSS [75].

MMP-12, also known as macrophage metalloelastase (MME), has a broad substrate specificity for matrix macromolecules, recognizing elastin, type IV collagen, fibronectin, or vitronectin. MMP-12 has been implicated in different pathological conditions including atherosclerosis, cancers, and skin diseases [76, 77]. In SSc patients, dermal fibroblasts expressed and released MMP-12 [78]. More recent studies reported that serum levels of MMP-12 were significantly increased in SSc patients, also correlating well with skin fibrosis, with dcSSc having higher levels of MMP-12 [79].

Lysyl oxidase (LOX) is an extracellular copper enzyme that cross-links collagen and elastin, thus stabilizing collagen fibrils. Consistent with its expression in the skin and fibroblasts in the context of SSc, the levels of LOX were elevated in the serum of SSc patients versus healthy controls. Further analysis revealed a correlation of LOX concentration with the mRSS in patients without lung fibrosis, indicating its specific correlation with skin fibrosis. Moreover, LOX levels were higher in SSc patients with dcSSc than in those with lcSSc, which may reflect a more advance fibrosis in diffuse subset of SSc [80].

In SSc patients, there is a strong relationship between inflammation and fibrosis supported by the upregulation of both, pro-inflammatory and pro-fibrotic markers in the serum as well as in skin. The role of different cytokines and chemokines has been analyzed in skin fibrosis of SSc in several studies. For instance, IL-6 and IL-10 serum levels were found to be elevated in SSc patients and significantly correlated with skin fibrosis assessed by mRSS [57]. However, recently, Codullo et al. confirmed that SSc patients expressed high level of IL-6 but did not find clear associations with mRSS or other clinical parameters [62].

CXCL4, largely viewed as a pro-inflammatory chemokine, in addition to its chemoattractant activity, regulates an array of immune cells, including T cells, monocytes, dendritic cells, as well as non-immune cells like endothelial cells. Recently, van Bon et al. used a proteomic approach and identified CXCL4 as a potential biomarker associated with multiple organ involvement in SSc. Circulating CXCL4 levels strongly correlated with the extent of skin fibrosis more with dcSSc subsets than lcSSc. In a prospective cohort study, elevated CXCL4 in the serum of SSc predicted a faster progression of skin fibrosis [81].

### Gene expression profiling

Gene expression profiling from skin biopsies is another interesting approach to identify biomarkers for skin fibrosis. Skin biopsies, although more difficult to obtain, allow for a more direct insight into the ongoing fibrotic reaction. Moreover, they can lead to the discovery of genes specific for different subsets of SSc and to predict if patients will develop more severe subset of the disease. For instance, in 2008, Milano et al. reported a 177-gene signature that was associated with severity of skin disease in diffuse subsets of SSc [82]. This identification not only allows for a better understanding of the disease pathogenesis but also provides important information for novel therapeutic targets.

TGF-β is one of the most potent pro-fibrotic cytokines in SSc and also one of the strongest stimulators for the differentiation of fibroblasts into activated myofibroblasts. Therefore, a group of researchers examined the expression of genes highly upregulated by TGF-β and found that some of these genes were highly correlated with the skin score. When they expanded their studies to interferon-regulated genes, they found that several of these genes also strongly correlated with the mRSS. Therefore, the combination of both TGF-β and IFN-regulated genes, namely COMP and thrombospondin-1 (TSP-1) (TGF-β regulated genes), and IFN-inducible 44 (IFI44) and sialoadhesin (Siglec-1) resulted in a particularly strong correlation with skin score [83].

Lysyl hydroxylase-2 (LH2), an enzyme involved in collagen biosynthesis, was found to be elevated in the skin biopsies and isolated fibroblasts of SSc patients and could represent a marker for the skin fibrotic activity in these patients. LH-2 overexpression was found to be accompanied by an associated increase in the Pyr cross-links present in the accumulated collagen in the SSc patients. These Pyr cross-links are critical for the mechanical stability and tensile strength of collagen [84] (Table 4).

## Biomarkers involved in SSc lung involvement

Pulmonary complications are common in SSc patients. They are most often manifested by the fibrotic interstitial lung disease (ILD), or pulmonary vascular disease leading to pulmonary arterial hypertension (PAH), or co-occurrence of both. Together, SSc-associated ILD and PAH are the major cause of disease-related mortality in SSc [85, 86].

### Interstitial lung disease

The majority of SSc patients have evidence of pulmonary fibrosis, based on autopsy and radiographic findings [87]. In the European League Against Rheumatism Scleroderma Trials and Research (EUSTAR) registry, pulmonary fibrosis appeared more common in diffuse than in limited SSc (53.4 vs 34.7 %) [9]. To detect ILD in SSc, a chest imaging using high-resolution computed tomography (HRCT) and pulmonary function tests (PFT), including the measurement of forced vital capacity (FVC) and diffusing capacity of lung for carbon monoxide (DLCO), are being used. A new quantitative HRCT has improved visual radiographic assessment of ILD but not yet widely available [88]. Moreover, repeated exposure to radiation can be detrimental. Although FVC and DLCO show correlation with HRCT and adequately measure lung function, they are not specific for ILD or the ongoing fibrotic process. Therefore, additional biomarkers that are more accessible, repeatable, and complement both HRCT and PFT are needed [89].

Several different studies have examined the use of the lung-epithelial-derived protein surfactant protein-D (SP-D) and glycoprotein Krebs von den Lungen-6 (KL-6). As reviewed by others previously, most studies showed increased serum SP-D and KL-6 and their correlation with decline in FVC and DLCO in SSc patients, however with varying degrees of correlation [90, 91]. A recent study showed that serum KL-6 correlated strongly with HRCT-fibrosis score, serum ATA titers, and correlated inversely with FVC and DLCO [53]. They found a moderate correlation of SP-D with HRCT-fibrosis score, but no significant association with other clinical parameters tested. Other studies also found correlation of serum KL-6, as well as COMP, with lung fibrosis [92]. Additionally, SSc patients with elevated SP-D and KL-6 had far more frequent ATA positivity and less frequent ACA compared to those with normal level [93]. Serum KL-6 also showed strong correlation with mRSS and disease activity index, indicating it to be a multipurpose biomarker candidate in SSc [53].

CCL18 is a chemokine produced by antigen presenting cells, particularly by alveolar macrophages in different interstitial lung diseases [94]. In SSc, the level of CCL18 was elevated in the bronchoalveolar lavage (BAL) fluid, lung, serum, and associated with lung involvement [95, 96]. One study showed moderate but significant negative correlation of serum CCL18 with DLCO and FVC in SSc [97]. In their retrospective cohort analysis, serum CCL-18 level was decreased in SSc patients having an improvement of pulmonary fibrosis (as measured by HRCT, PFT, and BAL analysis) and comparable to the decrease of KL-6 and SP-D [97]. Another study found a similar observation where serum CCL18 in SSc correlated with DLCO decline and total lung capacity (TLC) decline, and changes of TLC over a period of at least 6 months [95]. A longitudinal study of a 4-year period showed that a cut-off serum CCL18 value at 187 ng/ml is able to predict worsening ILD [98]. This was later reproduced in an independent cohort with a similar cut-off value and hazard ratios [99], but another study challenged this finding suggesting that correlation between CCL18 and changes in FVC could only be seen at a short term (1 year) but not at a longer period [100]. Interestingly, a recent microarray analysis of SSc-ILD lung showed that lung CCL18 RNA expression correlated with changes of HRCT-score FibMax and negatively correlated, although not strongly, with % predicted FVC [96].

van Bon et al. showed that chemokine CXCL4 was associated with lung disease manifestations in SSc [81]. Patients who had high circulating CXCL4 (>10 ng/ml) developed lung fibrosis earlier compared to those who had low CXCL4, as measured by >30 % decrease of FVC and HRCT. In the prospective cohort, patients with a high CXCL4 baseline showed a significantly faster decline in DLCO and a higher prevalence of HRCT-confirmed lung fibrosis. Earlier study has showed a significant increase of CXCL4 in BAL fluid from SSc patients exclusively those with ILD [101].

CXCL8 (IL-8) functions as the main chemotactic factor for neutrophils and other granulocytes. CXCL8 gene polymorphisms were associated with an increased susceptibility to SSc [102, 103]. Circulating CXCL8 level has been reported to be elevated in SSc patients [62, 104], but this was found not in all studies [60]. Two studies showed an increase of CXCL8 level in the BAL fluid from SSc patients that correlated with a more extensive lung fibrosis based on HRCT [92] and inversely correlated with DLCO, FVC, and TLC [105], but neither study measured circulating CXCL8. Other investigations using CXCL8 level in the serum of SSc patients showed no significant association with PFT or any future pulmonary involvement [62, 106]; whereas, one report showed serum CXCL8 association with DLCO decrease [104]. These findings suggest that CXCL8 level may more strongly reflect disease progression locally rather than systemically.

S100A8 (MRP-8, calgranulin A) and S100A9 (MRP-14, calgranulin B) are members of the S100 calcium-binding proteins. Together they form a complex, S100A8/9 (calprotectin, calgranulinA/B), that is able to modulate inflammatory processes mainly by binding to toll-like receptor 4 (TLR4) [107]. Earlier studies have shown increased of S100A8/9 or their homodimeric formats in the sera [108, 109], feces [110, 111], saliva [112], BAL fluids [92], and skin [109, 113] of SSc patients compared to healthy individuals. High level of S100A8/9 in the BAL fluid [92] and serum [109] in patients has been associated with an extensive lung fibrosis and ATA positivity, although no direct correlation to PFT was found. A recent proteomic analysis of serum samples from SSc patients also showed S100A8/9 to be increased particularly in lcSSc having lung fibrosis and ATA-positive patients [114]. As recent evidences point to the role of TLR4 signaling in fibrosis [115–117], S100A8/9 is a potentially interesting molecule to investigate further.

The serum level of GDF-15 has been shown to be strongly associated with multiple organ involvement in SSc especially in the lung. Serum GDF-15 level was higher in patients with ILD compared to those without, and its level in the serum negatively correlated with DLCO and FVC [64, 65]. Patients with high level of serum GDF-15 had a more frequent ATA and a less frequent ACA positivity [64]. Importantly, SSc patients with higher baseline GDF-15 level showed lower DLCO and worsened lung diseases severity score over a follow-up period of up to 30 months, suggesting its value as a predictive prognostic marker of lung function and fibrosis in SSc [65] (Table 5).

**Table 5:** Biomarker in SSc-ILD

Biomarker	Source	Association	Reference
SP-D and KL-6	Serum	HRCT, FVC, DLCO	[53, 92, 93, 118, 119]
CCL18	Serum, BAL, lung	HRCT, FVC, TLC, DLCO	[95, 96, 98–100]
CXCL4	Plasma, BAL	HRCT, FVC, DLCO	[81, 101]
CXCL8	Serum, BAL	HRCT, FVC, TLC, DLCO	[62, 92, 104–106]
S100A8/9	Serum, plasma, BAL	HRCT	[92, 109, 114]
COMP	Serum	HRCT	[92]
GDF-15	Serum	DLCO, FVC	[64, 65]

### Pulmonary arterial hypertension

Although PAH appears to be more frequent in the lcSSc than dcSSc (9.2 vs 5.9 % according to EULAR registry [9]), it can occur in all forms of SSc. Patients with SSc-PAH has a poor prognosis; therefore, an early detection of SSc-PAH and initiation of therapy are essential in disease management. Transthoracic echocardiography (TTE) of pulmonary artery systolic pressure is the most widely used screening tool for PAH; however, the method has considerable measurement variability and may not be sufficiently sensitive for the detection of early disease [120]. The more invasive right heart catheterization (RHC) is still the golden standard to confirm PAH in SSc patients.

N-terminal pro-brain natriuretic peptide (NT-proBNP), a biomarker of myocardial stress, has been intensively studied as a diagnostic marker for SSc-PAH. Serum NT-pro-BNP levels have been showed to strongly correlate with mean pulmonary arterial pressure (PAP) and pulmonary vascular resistance (PVR) [121]. They also showed association of NT-proBNP with severity of PAH and risk of mortality [121]. Later studies also showed similar correlation of NT-proBNP with increased PAP based on TTE and RHC, as well as negative correlation with DLCO and the presence of ATA [122–126]. More recently, NT-proBNP has been in used as a diagnostic marker in combination with other modalities including TTE and PFT. This approach, as reported by the DETECT study and others, gave an improved sensitivity and reduced missed diagnosis for early SSc-PAH as compared to the ESC/ERS guidelines [125, 127, 128]. However, it is important to note that this marker is not specific to PAH, as it may results from pulmonary venous hypertension or other cardiac dysfunction.

In search for surrogate marker for SSc-PAH, many investigators have looked into the molecules produced by or acting on the endothelium and have attempted to correlate them to hemodynamic and pulmonary function parameters. Markers of vascular injury such as vascular endothelial growth factor (VEGF), endothelin-1 (ET-1), and von Willebrand factor (vWF), as well as the soluble adhesion molecules ICAM-1 and VCAM-1 have been showed to be elevated in SSc sera and associated with PAH [129, 130]. Both circulating VEGF and ET-1 levels were found higher in SSc patients with PAH compared to those without and their level correlated positively with pulmonary arterial pressure [129, 131]. However, ET-1 association with pulmonary pressure or function was not found in another study [132]. Serum VEGF levels also correlated with decline of DLCO in cohorts of SSc patients without ILD [131] and in limited subtype [56]. The level of vWF in plasma of SSc patients was found to correlate with PAP, based on Doppler cardiography [133]. In a substudy from QUINs randomized placebo-controlled trial, baseline serum vWF antigen concentrations significantly related to disease activity, inversely correlated to %FVC and %DLCO at baseline, and were able to predict elevated PAP of >40 mmHg after 3 years, based on TTE [52]. However, another study did not find correlation between serum vWF and PFT measurements, perhaps due to a smaller cohort and a difference in statistical analysis [134]. In an 18-month prospective cohort, plasma vWF did not correlate with future changes in DLCO or skin score [81]. Furthermore, the use of vWF as a biomarker can be challenging since there is already a large variation in healthy individuals: ABO blood group, genetic polymorphism, and age are among the determinants [135, 136].

In addition to its association with skin and lung fibrosis, circulating CXCL4 levels were elevated in SSc patients who had evidence of PAH compared to those without, as determined on RHC [81]. In this study, high CXCL4 levels were also associated with an earlier development of PAH. In a transcriptome analysis of lung biopsies from idiopathic PAH patients, CXCL4 was one of the most highly and differentially expressed genes as compared to healthy controls [137]. As CXCL4 exerts angiostatic properties on pulmonary arterial endothelial cells [138], this suggests that CXCL4 might be involved in the pathophysiology of PAH in SSc.

As mentioned above, serum GDF-15 was associated with pulmonary fibrosis and impaired lung function. In SSc patients with PAH, the plasma level of GDF-15 was significantly higher as compared to those without [139]. Plasma concentrations of GDF-15 showed strong correlation with right ventricular systolic pressure (RVSP) on echocardiography, NT-proBNP plasma levels, and negative correlation with DLCO, but no correlation with any RHC-based hemodynamics [139]. Importantly, a ROC curve analysis showed that a plasma GDF-15 cut-off level at 125 pg/ml was able to identify SSc-PAH better than NT-proBNP at 473 pmol/L (93 % sensitivity and 88 % specificity vs 86 % sensitivity and 30 % specificity) and was able to predict mortality in SSc patients [139]. Later studies measuring serum GDF-15 in SSc patients had very few patients with PAH in their cohorts that make it difficult to interpret [64, 65]. In a cohort of patients with idiopathic PAH, elevated serum GDF-15 level was associated with right atrial pressure, wedge pressure, and serum NT-proBNP level [140]. They also showed potential prognostic value of serum GDF-15 as it was related to changes of serum NT-proBNP and venous oxygen saturation in their follow-up cohort [140] (Table 6).

**Table 6:** Biomarker in SSc-PAH

Biomarker	Source	Association	Reference
NT-proBNP	Serum	PAP, PVR, DLCO	[121–126]
VEGF	Serum	PAP, DLCO	[56, 131]
ET-1	Plasma	PAP	[130, 132]
vWF	Serum, plasma	PAP, FVC, DLCO	[52, 81, 133, 134]
Anti-AT1R and anti-ETAR	Serum	PAH development	[33, 34]
CXCL4	Plasma	PAH development	[81]
GDF-15	Plasma	RVSP, DLCO	[64, 65, 139]

## Novel approaches to identify biomarkers in SSc

The fast advancement of current molecular biology and biochemical techniques has moved research from the reductionist approach of studying one individual component at a time, towards a more holistic approach where multiple high-throughput omics layers—so called systems medicine—can be determined in clinically well-defined patient groups. Genomic-wide association studies, whole transcriptome, and proteome analysis have been performed in recent studies and yielded novel candidate biomarkers for SSc [141–143]. The use of more recent state-of-the-art technologies such as mass cytometry, that would enable us to phenotype immune compartments or other cells of interest in a great detail, are currently underway. The challenge of systemic multilayered large-data gathering approach is the complexity of big-data management, analysis, and interpretation. Computational models and computer learning algorithms are essential to answer specific research questions that hopefully lead investigators to the discovery of new biomarkers and understanding pathways.

## Conclusions

**Fig. 1:**
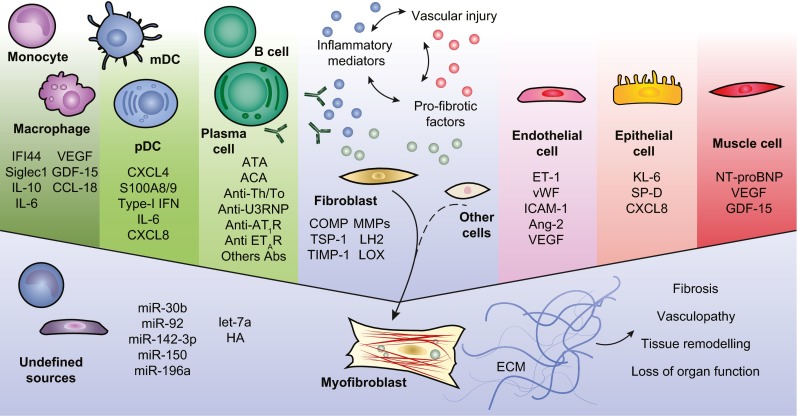
A schematic depiction of biomarkers in systemic sclerosis and their production by different cell types. Immune cells produce a large number of biomarkers that have been investigated in SSc, and their interaction with endothelial cells, fibroblasts, and other cell types may eventually lead to extracellular matrix (ECM) deposition and the progression of disease

Discoveries of new biomarkers and composite scores in SSc have supported the more conventional approaches in patients’ evaluation including mRSS, PFTs, RHC, and HRCT. For example, incorporating NT-proBNP to TTE and PFT measures has improved diagnosis of SSc-PAH significantly. Several biomarkers with clinically important multipurpose utility can give an added value, for example, both KL-6 and CXCL4 showed correlation with skin and lung involvement and predictive of future disease course. Many of these new promising biomarkers (see Fig. 1), however, still require validation and assessment in longitudinal cohorts or in clinical trials. More investigations in prognostic markers that can predict patients’ disease trajectory or differences in response to therapy are of urgent need. In the near future, systems medicine approaches including the true integration of multilayered data may provide more complete assessment of patients, novel biomarkers, understanding of disease, or even drug discovery and personalized therapy.

## References

[1] Gabrielli A, Avvedimento EV, Krieg T (2009). Scleroderma. N Engl J Med.

[2] Masi AT (1988). Classification of systemic sclerosis (scleroderma): relationship of cutaneous subgroups in early disease to outcome and serologic reactivity. J Rheumatol.

[3] Steen VD (2005). Autoantibodies in systemic sclerosis. Semin Arthritis Rheum.

[4] Walker JG, Fritzler MJ (2007). Update on autoantibodies in systemic sclerosis. Curr Opin Rheumatol.

[5] Graf SW, Hakendorf P, Lester S (2012). South Australian Scleroderma Register: autoantibodies as predictive biomarkers of phenotype and outcome. Int J Rheum Dis.

[6] Douvas AS, Achten M, Tan EM (1979). Identification of a nuclear protein (Scl-70) as a unique target of human antinuclear antibodies in scleroderma. J Biol Chem.

[7] Shero JH, Bordwell B, Rothfield NF, Earnshaw WC (1986). High titers of autoantibodies to topoisomerase I (Scl-70) in sera from scleroderma patients. Science.

[8] Basu D, Reveille JD (2005). Anti-scl-70. Autoimmunity.

[9] Walker UA, Tyndall A, Czirják L (2007). Clinical risk assessment of organ manifestations in systemic sclerosis: a report from the EULAR Scleroderma Trials and Research group database. Ann Rheum Dis.

[10] Reveille JD, Solomon DH (2003). Evidence-based guidelines for the use of immunologic tests: anticentromere, Scl-70, and nucleolar antibodies. Arthritis Rheum.

[11] Steen VD, Powell DL, Medsger TA (1988). Clinical correlations and prognosis based on serum autoantibodies in patients with systemic sclerosis. Arthritis Rheum.

[12] Hesselstrand R, Scheja A, Shen GQ (2003). The association of antinuclear antibodies with organ involvement and survival in systemic sclerosis. Rheumatology.

[13] Denton CP, Krieg T, Guillevin L (2012). Demographic, clinical and antibody characteristics of patients with digital ulcers in systemic sclerosis: data from the DUO Registry. Ann Rheum Dis.

[14] Hanke K, Dähnrich C, Brückner CS (2009). Diagnostic value of anti-topoisomerase I antibodies in a large monocentric cohort. Arthritis Res Ther.

[15] Earnshaw W, Bordwell B, Marino C, Rothfield N (1986). Three human chromosomal autoantigens are recognized by sera from patients with anti-centromere antibodies. J Clin Invest.

[16] Rothfield N, Whitaker D, Bordwell B (1987). Detection of anticentromere antibodies using cloned autoantigen CENP-B. Arthritis Rheum.

[17] Carwile LeRoy E, Black C, Fleischmajer R (1988). Scleroderma (systemic sclerosis): classification, subsets and pathogenesis. J Rheumatol.

[18] Koenig M, Dieudé M, Senécal JL (2008). Predictive value of antinuclear autoantibodies: the lessons of the systemic sclerosis autoantibodies. Autoimmun Rev.

[19] Mitri GM, Lucas M, Fertig N (2003). A comparison between anti-TH/To- and anticentromere antibody-positive systemic sclerosis patients with limited cutaneous involvement. Arthritis Rheum.

[20] Miyawaki S, Asanuma H, Nishiyama S, Yoshinaga Y (2005). Clinical and serological heterogeneity in patients with anticentromere antibodies. J Rheumatol.

[21] Akiyama Y, Tanaka M, Takeishi M (2000). Clinical, serological and genetic study in patients with CREST syndrome. Intern Med.

[22] Ferri C, Valentini G, Cozzi F (2002). Systemic sclerosis: demographic, clinical, and serologic features and survival in 1,012 Italian patients. Medicine (Baltimore).

[23] Chang M, Wang RJ, Yangco DT (1998). Analysis of autoantibodies against RNA polymerases using immunoaffinity-purifed RNA polymerase I, II, and III antigen in an enzyme-linked immunosorbent assay. Clin Immunol Immunopathol.

[24] Kuwana M, Okano Y, Pandey JP (2005). Enzyme-linked immunosorbent assay for detection of anti-RNA polymerase III antibody: analytical accuracy and clinical associations in systemic sclerosis. Arthritis Rheum.

[25] Kuwana M, Kaburaki J, Mimori T (1993). Autoantibody reactive with three classes of RNA polymerases in sera from patients with systemic sclerosis. J Clin Invest.

[26] Cavazzana I, Angela C, Paolo A (2009). Anti-RNA polymerase III antibodies: a marker of systemic sclerosis with rapid onset and skin thickening progression. Autoimmun Rev.

[27] Satoh M, Ajmani AK, Ogasawara T (1994). Autoantibodies to RNA polymerase II are common in systemic lupus erythematosus and overlap syndrome: specific recognition of the phosphorylated (IIO) form by a subset of human sera. J Clin Invest.

[28] Van Eenennaam H, Vogelzangs JHP, Lugtenberg D (2002). Identity of the RNase MRP- and RNase P-associated Th/To autoantigen. Arthritis Rheum.

[29] Okano Y, Medsger TA (1990). Autoantibody to Th ribonucleoprotein (nucleolar 7–2 RNA protein particle) in patients with systemic sclerosis. Arthritis Rheum.

[30] Fischer A, Pfalzgraf FJ, Feghali-Bostwick CA (2006). Anti-th/to-positivity in a cohort of patients with idiopathic pulmonary fibrosis. J Rheumatol.

[31] Kipnis RJ, Craft J, Hardin JA (1990). The analysis of antinuclear and antinucleolar autoantibodies of scleroderma by radioimmunoprecipitation assays. Arthritis Rheum.

[32] Aggarwal R, Lucas M, Fertig N (2009). Anti-U3 RNP autoantibodies in systemic sclerosis. Arthritis Rheum.

[33] Becker MO, Kill A, Kutsche M (2014). Vascular receptor autoantibodies in pulmonary arterial hypertension associated with systemic sclerosis. Am J Respir Crit Care Med.

[34] Riemekasten G, Philippe A, Näther M (2011). Involvement of functional autoantibodies against vascular receptors in systemic sclerosis. Ann Rheum Dis.

[35] Giovannetti A, Maselli A, Colasanti T (2013). Autoantibodies to estrogen receptor α in systemic sclerosis (SSc) as pathogenetic determinants and markers of progression. PLoS One.

[36] Fertig N, Domsic RT, Rodriguez-Reyna T (2009). Anti-U11/U12 RNP antibodies in systemic sclerosis: a new serologic marker associated with pulmonary fibrosis. Arthritis Care Res.

[37] Friedman RC, Farh KKH, Burge CB, Bartel DP (2009). Most mammalian mRNAs are conserved targets of microRNAs. Genome Res.

[38] Li H, Yang R, Fan X (2012). MicroRNA array analysis of microRNAs related to systemic scleroderma. Rheumatol Int.

[39] Zhu H, Li Y, Qu S (2012). MicroRNA expression abnormalities in limited cutaneous scleroderma and diffuse cutaneous scleroderma. J Clin Immunol.

[40] Honda N, Jinnin M, Kira-Etoh T (2013). MiR-150 down-regulation contributes to the constitutive type i collagen overexpression in scleroderma dermal fibroblasts via the induction of integrin β3. Am J Pathol.

[41] Honda N, Jinnin M, Kajihara I (2012). TGF-β-mediated downregulation of microRNA-196a contributes to the constitutive upregulated type I collagen expression in scleroderma dermal fibroblasts. J Immunol.

[42] Tanaka S, Suto A, Ikeda K (2013). Alteration of circulating miRNAs in SSc: miR-30b regulates the expression of PDGF receptor β. Rheumatology (Oxford).

[43] Makino K, Jinnin M, Hirano A (2013). The downregulation of microRNA let-7a contributes to the excessive expression of type I collagen in systemic and localized scleroderma. J Immunol.

[44] Sing T, Jinnin M, Yamane K (2012). MicroRNA-92a expression in the sera and dermal fibroblasts increases in patients with scleroderma. Rheumatology (Oxford).

[45] Makino K, Jinnin M, Kajihara I (2012). Circulating miR-142-3p levels in patients with systemic sclerosis. Clin Exp Dermatol.

[46] Valentini G, Bencivelli W, Bombardieri S (2003). European Scleroderma Study Group to define disease activity criteria for systemic sclerosis. III. Assessment of the construct validity of the preliminary activity criteria. Ann Rheum Dis.

[47] Valentini G, Silman AJ, Veale D (2003). Assessment of disease activity. Clin Exp Rheumatol.

[48] Young-Min SA, Beeton C, Laughton R (2001). Serum TIMP-1, TIMP-2, and MMP-1 in patients with systemic sclerosis, primary Raynaud’s phenomenon, and in normal controls. Ann Rheum Dis.

[49] Scheja A, Akesson A, Hørslev-Petersen K (1992). Serum levels of aminoterminal type III procollagen peptide and hyaluronan predict mortality in systemic sclerosis. Scand J Rheumatol.

[50] Nagy Z, Czirják L (2005). Increased levels of amino terminal propeptide of type III procollagen are an unfavourable predictor of survival in systemic sclerosis. Clin Exp Rheumatol.

[51] Abignano G, Cuomo G, Buch MH (2014). The enhanced liver fibrosis test: a clinical grade, validated serum test, biomarker of overall fibrosis in systemic sclerosis. Ann Rheum Dis.

[52] Barnes T, Gliddon A, Doré CJ (2012). Baseline vWF factor predicts the development of elevated pulmonary artery pressure in systemic sclerosis. Rheumatology (Oxford).

[53] Bonella F, Volpe A, Caramaschi P (2011). Surfactant protein D and KL-6 serum levels in systemic sclerosis: correlation with lung and systemic involvement. Sarcoidosis Vasc Diffuse Lung Dis.

[54] Gheita TA, Hussein H (2012). Cartilage oligomeric matrix protein (COMP) in systemic sclerosis (SSc): role in disease severity and subclinical rheumatoid arthritis overlap. Joint Bone Spine.

[55] Michalska-Jakubus M, Kowal-Bielecka O, Chodorowska G (2011). Angiopoietins-1 and -2 are differentially expressed in the sera of patients with systemic sclerosis: high angiopoietin-2 levels are associated with greater severity and higher activity of the disease. Rheumatology.

[56] Dunne JV, Keen KJ, Van Eeden SF (2013). Circulating angiopoietin and Tie-2 levels in systemic sclerosis. Rheumatol Int.

[57] Sato S, Hasegawa M, Takehara K (2001). Serum levels of interleukin-6 and interleukin-10 correlate with total skin thickness score in patients with systemic sclerosis. J Dermatol Sci.

[58] Scala E, Pallotta S, Frezzolini A (2004). Cytokine and chemokine levels in systemic sclerosis: relationship with cutaneous and internal organ involvement. Clin Exp Immunol.

[59] De Lauretis A, Sestini P, Pantelidis P (2013). Serum interleukin 6 is predictive of early functional decline and mortality in interstitial lung disease associated with systemic sclerosis. J Rheumatol.

[60] Gourh P, Arnett FC, Assassi S (2009). Plasma cytokine profiles in systemic sclerosis: associations with autoantibody subsets and clinical manifestations. Arthritis Res Ther.

[61] Jurisic Z, Martinovic-Kaliterna D, Marasovic-Krstulovic D (2013). Relationship between interleukin-6 and cardiac involvement in systemic sclerosis. Rheumatology (Oxford).

[62] Codullo V, Baldwin HM, Singh MD (2011). An investigation of the inflammatory cytokine and chemokine network in systemic sclerosis. Ann Rheum Dis.

[63] Sfrent-Cornateanu R, Mihai C, Balan S (2006). The IL-6 promoter polymorphism is associated with disease activity and disability in systemic sclerosis. J Cell Mol Med.

[64] Yanaba K, Asano Y, Tada Y (2012). Clinical significance of serum growth differentiation factor-15 levels in systemic sclerosis: association with disease severity. Mod Rheumatol.

[65] Lambrecht S, Smith V, De Wilde K (2014). Growth differentiation factor 15, a marker of lung involvement in systemic sclerosis, is involved in fibrosis development but is not indispensable for fibrosis development. Arthritis Rheum.

[66] Czirják L, Foeldvari I, Müller-Ladner U (2008). Skin involvement in systemic sclerosis. Rheumatology (Oxford).

[67] Farina G, Lemaire R, Korn JH, Widom RL (2006). Cartilage oligomeric matrix protein is overexpressed by scleroderma dermal fibroblasts. Matrix Biol.

[68] Farina G, Lemaire R, Pancari P (2009). Cartilage oligomeric matrix protein expression in systemic sclerosis reveals heterogeneity of dermal fibroblast responses to transforming growth factor beta. Ann Rheum Dis.

[69] Hesselstrand R, Kassner A, Heinegård D, Saxne T (2008). COMP: a candidate molecule in the pathogenesis of systemic sclerosis with a potential as a disease marker. Ann Rheum Dis.

[70] Wynn TA (2007). Common and unique mechanisms regulate fibrosis in various fibroproliferative diseases. J Clin Invest.

[71] Ahrens D, Koch AE, Pope RM (1996). Expression of matrix metalloproteinase 9 (96-kd gelatinase B) in human rheumatoid arthritis. Arthritis Rheum.

[72] Liu Y, Zheng M, Yin W, Zhang B (2004). Relationship of serum levels of HGF and MMP-9 with disease activity of patients with systemic lupus erythematosus. Zhejiang Da Xue Xue Bao Yi Xue Ban.

[73] Pardo A, Selman M (2012). Role of matrix metalloproteases in idiopathic pulmonary fibrosis. Fibrogenesis Tissue Repair.

[74] Venkateshwari A, Sri Manjari K, Krishnaveni D (2011). Role of plasma MMP 9 levels in the pathogenesis of chronic pancreatitis. Indian J Clin Biochem.

[75] Kim W-U, Min S-Y, Cho M-L (2005). Elevated matrix metalloproteinase-9 in patients with systemic sclerosis. Arthritis Res Ther.

[76] Said AH, Raufman J-P, Xie G (2014). The role of matrix metalloproteinases in colorectal cancer. Cancers (Basel).

[77] Pardo A, Selman M (2006). Matrix metalloproteases in aberrant fibrotic tissue remodeling. Proc Am Thorac Soc.

[78] Serratì S, Cinelli M, Margheri F (2006). Systemic sclerosis fibroblast inhibit in vitro angiogenesis by MMP-12-dependent cleavage of the endothelial cell urokinase receptor. J Pathol.

[79] Manetti M, Guiducci S, Romano E (2012). Increased serum levels and tissue expression of matrix metalloproteinase-12 in patients with systemic sclerosis: correlation with severity of skin and pulmonary fibrosis and vascular damage. Ann Rheum Dis.

[80] Rimar D, Rosner I, Nov Y (2014). Brief report: lysyl oxidase is a potential biomarker of fibrosis in systemic sclerosis. Arthritis Rheumatol.

[81] Van Bon L, Affandi AJ, Broen J (2014). Proteome-wide analysis and CXCL4 as a biomarker in systemic sclerosis. N Engl J Med.

[82] Milano A, Pendergrass SA, Sargent JL (2008). Molecular subsets in the gene expression signatures of scleroderma skin. PLoS One.

[83] Farina G, Lafyatis D, Lemaire R (2010). A four-gene biomarker predicts skin disease in patients with diffuse cutaneous systemic sclerosis. Arthritis Rheum.

[84] Brinckmann J, Kim S, Wu J (2005). Interleukin 4 and prolonged hypoxia induce a higher gene expression of lysyl hydroxylase 2 and an altered cross-link pattern: important pathogenetic steps in early and late stage of systemic scleroderma?. Matrix Biol.

[85] Steen VD, Medsger TA (2007). Changes in causes of death in systemic sclerosis, 1972–2002. Ann Rheum Dis.

[86] Wells AU, Steen V, Valentini G (2009). Pulmonary complications: one of the most challenging complications of systemic sclerosis. Rheumatology (Oxford).

[87] Solomon JJ, Olson AL, Fischer A (2013). Scleroderma lung disease. Eur Respir Rev.

[88] Tashkin DP, Volkmann ER, Tseng C-HC-H, et al. (2014) Relationship between quantitative radiographic assessments of interstitial lung disease and physiological and clinical features of systemic sclerosis. Ann Rheum Dis 0:1–8. doi:10.1136/annrheumdis-2014-206076 10.1136/annrheumdis-2014-206076 25452309

[89] Abignano G, Buch M, Emery P, Del Galdo F (2011). Biomarkers in the management of scleroderma: an update. Curr Rheumatol Rep.

[90] Lafyatis R (2012). Application of biomarkers to clinical trials in systemic sclerosis. Curr Rheumatol Rep.

[91] Lota HK, Renzoni EA (2012). Circulating biomarkers of interstitial lung disease in systemic sclerosis. Int J Rheum.

[92] Hesselstrand R, Wildt M, Bozovic G (2013). Biomarkers from bronchoalveolar lavage fluid in systemic sclerosis patients with interstitial lung disease relate to severity of lung fibrosis. Respir Med.

[93] Yanaba K, Hasegawa M, Takehara K, Sato S (2004). Comparative study of serum surfactant protein-D and KL-6 concentrations in patients with systemic sclerosis as markers for monitoring the activity of pulmonary fibrosis. J Rheumatol.

[94] Cai M, Bonella F, He X (2013). CCL18 in serum, BAL fluid and alveolar macrophage culture supernatant in interstitial lung diseases. Respir Med.

[95] Prasse A, Pechkovsky DV, Toews GB (2007). CCL18 as an indicator of pulmonary fibrotic activity in idiopathic interstitial pneumonias and systemic sclerosis. Arthritis Rheum.

[96] Christmann RB, Sampaio-Barros P, Stifano G (2014). Association of interferon- and transforming growth factor β-regulated genes and macrophage activation with systemic sclerosis-related progressive lung fibrosis. Arthritis Rheumatol.

[97] Kodera M, Hasegawa M, Komura K (2005). Serum pulmonary and activation-regulated chemokine/CCL18 levels in patients with systemic sclerosis: a sensitive indicator of active pulmonary fibrosis. Arthritis Rheum.

[98] Tiev KP, Hua-Huy T, Kettaneh A (2011). Serum CC chemokine ligand-18 predicts lung disease worsening in systemic sclerosis. Eur Respir J.

[99] Schupp J, Becker M, Günther J (2014). Serum CCL18 is predictive for lung disease progression and mortality in systemic sclerosis. Eur Respir J.

[100] Elhaj M, Charles J, Pedroza C (2013). Can serum surfactant protein d or cc-chemokine ligand 18 predict outcome of interstitial lung disease in patients with early systemic sclerosis?. J Rheumatol.

[101] Kowal-Bielecka O, Kowal K, Lewszuk A (2005). Beta thromboglobulin and platelet factor 4 in bronchoalveolar lavage fluid of patients with systemic sclerosis. Ann Rheum Dis.

[102] Eun BL, Zhao J, Jeong YK (2007). Evidence of potential interaction of chemokine genes in susceptibility to systemic sclerosis. Arthritis Rheum.

[103] Salim PH, Jobim M, Bredemeier M (2012). Combined effects of CXCL8 and CXCR2 gene polymorphisms on susceptibility to systemic sclerosis. Cytokine.

[104] Furuse S, Fujii H, Kaburagi Y (2003). Serum concentrations of the CXC chemokines interleukin 8 and growth-regulated oncogene-alpha are elevated in patients with systemic sclerosis. J Rheumatol.

[105] Schmidt K, Martinez-Gamboa L, Meier S (2009). Bronchoalveolar lavage fluid cytokines and chemokines as markers and predictors for the outcome of interstitial lung disease in systemic sclerosis patients. Arthritis Res Ther.

[106] Hasegawa M, Asano Y, Endo H (2012). Serum chemokine levels as prognostic markers in patients with early systemic sclerosis: a multicenter, prospective, observational study. Mod Rheumatol.

[107] Vogl T, Tenbrock K, Ludwig S (2007). Mrp8 and Mrp14 are endogenous activators of Toll-like receptor 4, promoting lethal, endotoxin-induced shock. Nat Med.

[108] Kuruto R, Nozawa R, Takeishi K (1990). Myeloid calcium binding proteins: expression in the differentiated HL-60 cells and detection in sera of patients with connective tissue diseases. J Biochem.

[109] Xu X, Wu WY, Tu WZ (2013). Increased expression of S100A8 and S100A9 in patients with diffuse cutaneous systemic sclerosis. A correlation with organ involvement and immunological abnormalities. Clin Rheumatol.

[110] Andréasson K, Scheja A, Saxne T (2011). Faecal calprotectin: a biomarker of gastrointestinal disease in systemic sclerosis. J Intern Med.

[111] Andréasson K, Saxne T, Scheja A (2014). Faecal levels of calprotectin in systemic sclerosis are stable over time and are higher compared to primary Sjogren’s syndrome and rheumatoid arthritis. Arthritis Res Ther.

[112] Giusti L, Bazzichi L, Baldini C (2007). Specific proteins identified in whole saliva from patients with diffuse systemic sclerosis. J Rheumatol.

[113] Nikitorowicz-Buniak J, Shiwen X, Denton CP (2014). Abnormally differentiating keratinocytes in the epidermis of systemic sclerosis patients show enhanced secretion of CCN2 and S100A9. J Investig Dermatol.

[114] Van Bon L, Cossu M, Loof A et al (2014) Proteomic analysis of plasma identifies the toll-like receptor agonists S100A8/A9 as a novel possible marker for systemic sclerosis phenotype. Ann Rheum Dis 73:1585–1589. doi:10.1136/annrheumdis-2013-205013 10.1136/annrheumdis-2013-205013 24718960

[115] Bhattacharyya S, Kelley K, Melichian D (2012). Toll-like receptor 4 signaling augments transforming growth factor-ß responses: a novel mechanism for maintaining and amplifying fibrosis in scleroderma. Am J Pathol.

[116] Stifano G, Affandi AJ, Mathes AL (2014). Chronic toll-like receptor 4 stimulation in skin induces inflammation, macrophage activation, transforming growth factor beta signature gene expression, and fibrosis. Arthritis Res Ther.

[117] Takahashi T, Asano Y, Ichimura Y (2015). Amelioration of tissue fibrosis by toll-like receptor 4 knockout in murine models of systemic sclerosis. Arthritis Rheumatol.

[118] Asano Y, Ihn H, Yamane K (2001). Clinical significance of surfactant protein D as a serum marker for evaluating pulmonary fibrosis in patients with systemic sclerosis. Arthritis Rheum.

[119] Hant FN, Ludwicka-Bradley A, Wang H-J (2009). Surfactant protein D and KL-6 as serum biomarkers of interstitial lung disease in patients with scleroderma. J Rheumatol.

[120] Mukerjee D, St George D, Knight C (2004). Echocardiography and pulmonary function as screening tests for pulmonary arterial hypertension in systemic sclerosis. Rheumatology (Oxford).

[121] Williams MH, Handler CE, Akram R (2006). Role of N-terminal brain natriuretic peptide (N-TproBNP) in scleroderma-associated pulmonary arterial hypertension. Eur Heart J.

[122] Choi HJ, Shin YK, Lee HJ (2008). The clinical significance of serum N-terminal pro-brain natriuretic peptide in systemic sclerosis patients. Clin Rheumatol.

[123] Cavagna L, Caporali R, Klersy C (2010). Comparison of brain natriuretic peptide (BNP) and NT-proBNP in screening for pulmonary arterial hypertension in patients with systemic sclerosis. J Rheumatol.

[124] Elshamy HA, Ibrahim SE, Farouk HM (2011). N-terminal pro-brain natriuretic peptide in systemic sclerosis: new insights. Eur J Dermatol.

[125] Thakkar V, Stevens WM, Prior D (2012). N-terminal pro-brain natriuretic peptide in a novel screening algorithm for pulmonary arterial hypertension in systemic sclerosis: a case–control study. Arthritis Res Ther.

[126] Oravec RM, Bredemeier M, Laurino CC (2010). NT-proBNP levels in systemic sclerosis: association with clinical and laboratory abnormalities. Clin Biochem.

[127] Coghlan JG, Denton CP, Grünig E (2014). Evidence-based detection of pulmonary arterial hypertension in systemic sclerosis: the DETECT study. Ann Rheum Dis.

[128] Allanore Y, Borderie D, Avouac J (2008). High N-terminal pro-brain natriuretic peptide levels and low diffusing capacity for carbon monoxide as independent predictors of the occurrence of precapillary pulmonary arterial hypertension in patients with systemic sclerosis. Arthritis Rheum.

[129] Schmidt J, Launay D, Soudan B (2007). Assessment of plasma endothelin level measurement in systemic sclerosis. Rev Med Interne.

[130] Pendergrass SA, Hayes E, Farina G (2010). Limited systemic sclerosis patients with pulmonary arterial hypertension show biomarkers of inflammation and vascular injury. PLoS One.

[131] Papaioannou AI, Zakynthinos E, Kostikas K (2009). Serum VEGF levels are related to the presence of pulmonary arterial hypertension in systemic sclerosis. BMC Pulm Med.

[132] Morelli S, Ferri C, Di Francesco L (1995). Plasma endothelin-1 levels in patients with systemic sclerosis: influence of pulmonary or systemic arterial hypertension. Ann Rheum Dis.

[133] Scheja A, Akesson A, Geborek P (2001). Von Willebrand factor propeptide as a marker of disease activity in systemic sclerosis (scleroderma). Arthritis Res.

[134] Iannone F, Riccardi MT, Guiducci S (2008). Bosentan regulates the expression of adhesion molecules on circulating T cells and serum soluble adhesion molecules in systemic sclerosis-associated pulmonary arterial hypertension. Ann Rheum Dis.

[135] Jenkins PV, O’Donnell JS (2006). ABO blood group determines plasma von Willebrand factor levels: a biologic function after all?. Transfusion.

[136] Van Loon JE, Kavousi M, Leebeek FWG (2012). von Willebrand factor plasma levels, genetic variations and coronary heart disease in an older population. J Thromb Haemost.

[137] Rajkumar R, Konishi K, Richards TJ (2010). Genomewide RNA expression profiling in lung identifies distinct signatures in idiopathic pulmonary arterial hypertension and secondary pulmonary hypertension. Am J Physiol Heart Circ Physiol.

[138] Zabini D, Nagaraj C, Stacher E (2012). Angiostatic factors in the pulmonary endarterectomy material from chronic thromboembolic pulmonary hypertension patients cause endothelial dysfunction. PLoS One.

[139] Meadows CA, Risbano MG, Zhang L (2011). Increased expression of growth differentiation factor-15 in systemic sclerosis-associated pulmonary arterial hypertension. Chest.

[140] Nickel N, Kempf T, Tapken H (2008). Growth differentiation factor-15 in idiopathic pulmonary arterial hypertension. Am J Respir Crit Care Med.

[141] Korman BD, Criswell LA (2015). Recent advances in the genetics of systemic.

[142] Bălănescu P, Lădaru A, Bălănescu E (2014). Systemic sclerosis biomarkers discovered using mass-spectrometry-based proteomics: a systematic review. Biomarkers.

[143] Limpers A, van Royen-Kerkhof A, van Roon JA (2014). Overlapping gene expression profiles indicative of antigen processing and the interferon pathway characterize inflammatory fibrotic skin diseases. Expert Rev Clin Immunol.

